# Physiotherapists’ knowledge and experiences of palliative care in neonatal intensive care units: a qualitative study

**DOI:** 10.1590/1984-0462/2026/44/2025359

**Published:** 2026-10-26

**Authors:** Lorena Oliveira Bezerra Morais, Adriana Valongo Zani, Fernanda Pegoraro de Godoi Melo, Natália Trindade, Vanessa Suziane Probst

**Affiliations:** a Universidade Estadual de Londrina, Londrina, PR, Brazil.

**Keywords:** Palliative care, Physiotherapy, Neonatology, Cuidados paliativos, Fisioterapia, Neonatologia

## Abstract

**Objective:**

To explore physiotherapists’ knowledge and experiences regarding neonatal palliative care in the neonatal intensive care unit setting.

**Methods:**

A qualitative study using content analysis as a methodological reference. The sample consisted of 12 physiotherapists working in intensive care units in two cities in the northern region of Paraná. Data were collected from January to March 2024 through semi-structured interviews and organized using the IRaMuTeQ^®^ software.

**Results:**

The analyses led to the identification of five categories: 1) Neonatal Palliative Care: Comfort and Quality of Life; 2) Emotional Aspects and Reflections on Neonatal Palliative Care; 3) Challenges in and Strategies for Family Communication in Palliative Care; 4) Physiotherapeutic Practice in Hospital and Home Settings within the Context of Neonatal Palliative Care; and 5) Challenges and Strategies for Neonatal Palliative Care Implementation.

**Conclusions:**

Physiotherapists demonstrate relevant knowledge of neonatal palliative care, although institutional gaps and the need for further training remain. Expanding their participation in palliative care discussions may support the development of guidelines, strengthen training, and improve the quality of care.

## RESUMO

**Objetivo:**

Explorar o conhecimento e experiências dos fisioterapeutas sobre cuidados paliativos neonatais no contexto da unidade de terapia intensiva neonatal (UTIN).

**Métodos:**

Estudo qualitativo utilizando a análise de conteúdo como referência metodológica. A amostra foi composta por 12 fisioterapeutas que trabalham em UTINs em duas cidades da região norte do Paraná. Os dados foram coletados de janeiro a março de 2024 por meio de entrevistas semiestruturadas e organizados no software IRaMuTeQ®.

**Resultados:**

As análises levaram à identificação de cinco categorias: 1) Cuidados Paliativos Neonatais: Conforto e Qualidade de Vida; 2) Aspectos Emocionais e Reflexões sobre Cuidados Paliativos Neonatais; 3) Desafios e Estratégias na Comunicação com a Família em Cuidados Paliativos; 4) Prática Fisioterapêutica em Ambientes Hospitalares e Domiciliares no Contexto dos Cuidados Paliativos Neonatais e 5) Desafios e Estratégias para a Implementação dos Cuidados Paliativos Neonatais.

**Conclusões:**

Os fisioterapeutas demonstram conhecimento relevante em cuidados paliativos neonatais, embora ainda existam lacunas institucionais e necessidade de maior capacitação. A ampliação de sua participação nas discussões em cuidados paliativos pode contribuir para o desenvolvimento de diretrizes, fortalecer a formação profissional e melhorar a qualidade da assistência.

## INTRODUCTION

 The World Health Organization (WHO) defines neonatal palliative care as a multidisciplinary approach to improving quality of life for patients and their families by preventing and relieving physical, psychological, social, and spiritual suffering.^1^ In some cases, intensive care may be initiated before a confirmed postnatal diagnosis; however, when a definitive prenatal diagnosis of a life-limiting condition is established, a comfort-focused palliative care plan may be implemented.^2,3^ The heterogeneity of clinical conditions results in care being delivered across multiple settings, including neonatal intensive care units (NICUs), hospital wards, outpatient services, and home care.^4^

 In this context, individualized care plans are essential to guiding clinical decision-making and postnatal management, while respecting the values and preferences of families who choose to continue the pregnancy to its natural course.^5^ Furthermore, advances in intensive care have heightened the need to integrate palliative care within the NICU.^6^ Nevertheless, compared with the field of adult palliative care, neonatology still lacks a robust and comprehensive conceptual framework to support practice and research.^7^

 Interdisciplinary collaboration is a cornerstone of neonatal palliative care.^8^ The provision of palliative care in the NICU faces challenges, requiring continuous professional training. In this scenario, physiotherapy stands out as an essential part of the interdisciplinary team, contributing to the quality of life of newborns with life-limiting conditions by controlling symptoms, preventing complications, and promoting comfort.^9^ In this collaborative environment, healthcare professionals often function as mediators between the medical team and families, ensuring that clinical decisions and emotional support strategies are tailored to the unique needs of each case.^10^ The establishment of a multidisciplinary quality improvement team is crucial in guiding end-of-life care for the most vulnerable pediatric population, those in the NICU.^11^

 Physiotherapy uses physical resources, techniques, and symptom management exercises to prevent complications and thereby improve quality of life. However, physiotherapists are often less involved in palliative care for this population. Current evidence suggests that physiotherapists remain under-represented in neonatal palliative care.^12^ This limited participation may restrict the potential contributions of physiotherapeutic interventions to newborn care and support for their families. There is a clear lack of studies that provide in-depth analysis of the physiotherapist’s role within multidisciplinary palliative care teams in NICUs. While qualitative research exists in neonatal palliative care involving professions such as nursing,^13^ no such studies focus specifically on physiotherapy, underscoring the pioneering nature of this work. 

 In this way, it is essential to examine the role of physiotherapists in the NICU, considering their knowledge in neonatal palliative care, clinical experience, and interactions with the multidisciplinary team. Therefore, the present study aimed to explore physiotherapists’ knowledge and experiences concerning neonatal palliative care in NICUs. 

## METHOD

 This qualitative study employed Bardin’s content analysis.^14^ The study was approved by the University’s Research Ethics Committee (CAAE: 73844923.9.0000.5231) and conducted in accordance with the Consolidated Criteria for Reporting Qualitative Research (COREQ) to ensure methodological rigor.^15^ The sample comprised physiotherapists working in the NICU of hospitals in two cities in northern Paraná. After the Research Ethics Committee’s approval, the physiotherapy team coordinator provided the contact details of physiotherapists. Participants were recruited through an invitation sent via the WhatsApp application. After consent, a semi-structured interview was scheduled with each participant. 

 The inclusion criteria comprised physiotherapists with at least six months of professional experience who had been involved in the care of newborns requiring palliative care within their respective clinical settings. Professionals who were on vacation or leave during the data collection period were excluded. All participants provided their consent by signing the Informed Consent Form (ICF). 

 The institution in city A (ICA), accredited by the Unified Health System (SUS), is a referral center for high-risk pregnancies with a maternal–infant unit comprising 10 NICU beds and 14 neonatal intermediate care beds. The physiotherapy team included six professionals working rotating shifts, providing 18 hours of daily coverage, in addition to two residents during daytime shifts. The institution in city B (ICB), also SUS-accredited and operating as a philanthropic hospital, provides care for public, private, and self-paying patients. Its mixed NICU has 20 beds, with a physiotherapy team comprised of nine professionals who work rotating shifts and also ensure 18 hours of daily coverage. 

 Data were collected using a semi-structured instrument divided into two parts. The first gathered participants’ demographic and professional characteristics. The second comprised eight open-ended questions that explored physiotherapists’ understanding and previous experiences with neonatal palliative care, perceived challenges in clinical practice and family support, the role of physiotherapy in different care settings, participation in multidisciplinary decision-making, the existence of institutional protocols, and strategies to improve care. Participants were also invited to provide additional comments. 

 Interviews were conducted individually in private rooms in ICA and ICB, outside the participants’ workplaces, to ensure comfort and confidentiality. Each interview lasted approximately 40 minutes and was conducted between January and March 2024. 

 The interview guide was pilot tested with one physiotherapist to assess its suitability for the study objectives. This participant was excluded from the final sample. All interviews were conducted by the principal researcher, a physiotherapist trained in qualitative research methods. Interviews were audio-recorded using the recorder application on an Apple iPhone 14 Pro Max^®^ (128 GB) running iOS 16 (2023). 

 Recordings were transcribed verbatim using Microsoft Word 2019 (Microsoft Corporation, Redmond, WA, USA) and analyzed in detail. To ensure anonymity, participants were identified by the letter “I” (informant), followed by ICA or ICB and a sequential interview number. As the principal researcher was a physiotherapist with previous neonatal intensive care experience, reflexivity was maintained throughout data collection and analysis to minimize potential bias, with careful attention to participants’ narratives and the suspension of prior assumptions. 

 Saturation was achieved when participants’ narratives reached a high level of recurrence and homogeneity, with no new relevant elements identified for the development of subsequent categories.^14^

 The transcribed interviews were analyzed through pre-analysis, material exploration, data treatment, and thematic categorization. Pre-analysis consisted of a floating reading and identification of recording units, which were grouped by similarity. Codes representing key ideas were then generated and organized into thematic categories aligned with the study objectives. Figures illustrating word prominence within each category were produced using IRaMuTeQ^®^ software (version 0.8 alpha 7; R version 4.5.1). The software was used to support text corpus organization. The analytical process involved the principal investigator, supervisor, and co-supervisor, using researcher triangulation. Data were independently coded and categorized, and interpretations were compared and refined through discussion to reach consensus, reducing bias and improving analytical consistency. 

 In addition, textual data were analyzed using the Similarity Analysis method, which is grounded in graph theory. The hierarchical relationships between terms reveal conceptual proximities, enabling the identification of word co-occurrences. This analysis produces a visual representation known as a similarity tree.^16^ Researcher triangulation was applied during the analytical process, with two researchers independently coding and categorizing the data, followed by comparison and consensus to reduce bias and ensure analytical consistency. Similarity analysis was interpreted through graphical representation, in which more frequent terms occupied central positions, and links indicated the strength of associations within the text corpus. 

## RESULTS

 Of the eight physiotherapists employed at the institution in ICA, one declined to participate, and two were excluded for being residents who did not meet the study’s eligibility criteria. At the institution in ICB, two of the nine physiotherapists were excluded because they were on vacation. Therefore, the final study sample comprised twelve physiotherapists (Table 1). 

**Table 1.** Characteristics of the physiotherapists participating in the study according to institution.

Variables		ICA (n=5)	ICB (n=7)
Age (years)		31±1.5	34±5.5
Workload (hours/week)		30	30
Professional experience (years)		8±1.8	11±6.1
Neonatal experience (years)		6±2.5	8±5.6
Educational background			
	Specialization, n (%)	5 (100)	7 (100)
	Master’s degree, n (%)	3 (60)	2 (29)
	Doctoral degree (PhD), n (%)	1 (20)	0 (0)

ICA: Institution in city A; ICB: Institution in city B.

Source: Created by the authors.

 Regarding category 1, *Neonatal Palliative Care: Comfort and Quality of Life*, participants described neonatal palliative care as the implementation of individualized strategies focused on the newborn’s needs, to relieve pain, optimize ventilatory support when appropriate, and minimize suffering. It also seeks to avoid unnecessary prolongation of intensive care unit stay. A key aspect identified was the reduced use of medications to revert life-limiting conditions, prioritizing comfort and quality of life over aggressive or non-beneficial treatments. Family comfort and well-being were also considered essential components of care. As one participant explained, “...*it would be when we promote maximum comfort for the newborn, without excessive investment in relation to medications or actions within a neonatal unit*.” *(ICA3)*

 As for category 2, *Emotional Aspects and Reflections on Neonatal Palliative Care*, physiotherapists reported experiencing a range of emotional responses when addressing neonatal palliative care in their practice. Feelings such as sadness, pity, and distress related to the newborn’s prognosis and the suffering of families, particularly parents, were commonly described as impactful in the NICU context. One participant said,“...*we feel sorry for the parents and the baby having to live through that situation*.” (ICA1), while another stated that “...*families have different ways of coping; some families deal better or worse, it all depends on various factors, but witnessing their suffering is very upsetting*.” (ICA4), and a third added that “...*I feel a mix of emotions. I don’t know if it’s because I am a mother. However, over the past few years, I’ve started studying and following palliative care closely. So, it has become a little softer, in the sense that you realize it’s not about abandoning patients, but rather about assisting them in a dignified way during their last moments*.” (ICB7). Differences in emotional responses were observed, particularly among physiotherapists who are mothers, for whom the emotional impact of neonatal palliative care appeared more intense and personally significant. 

 Regarding category 3, *Challenges in and Strategies for Family Communication in Palliative Care*, family acceptance of a poor prognosis and the healthcare team’s knowledge were identified as key factors influencing communication with parents of neonatal patients. Understanding families’ faith and belief systems was also considered essential because respecting these aspects supports a more sensitive and appropriate approach to care. Effective communication between healthcare professionals and families was recognized as fundamental to humanized care. However, limited knowledge of palliative care was reported as a barrier, highlighting the need for continuous education and a structured interdisciplinary approach. One participant stated that “...*In the NICU, extensive communication with parents is required. But the most difficult thing is the acceptance of the family and understanding of the clinical situation*.” (ICA5). Another concluded that “...*There are difficulties, and one way to overcome them would be to understand the family’s spirituality, beliefs, and expectations. By identifying these before approaching the family, the team may provide more appropriate care for each case*.” (ICA4). 

 As for category 4, *Physiotherapeutic Practice in Hospital and Home Settings within Neonatal Palliative Care*, physiotherapeutic interventions in intensive care and home settings were considered similar in several aspects, particularly airway clearance, secretion management, and the prevention of respiratory, motor, and musculoskeletal complications. In the NICU, emphasis was placed on structural aspects of care, mechanical ventilation management, the kangaroo mother care method, and neonatal clinical instability. In contrast, home care focused on motor development, health education, and maintaining infant stability within the family context. As one participant explained, “...*Most of our patients, being newborns and infants in palliative care, are on invasive ventilation. In this context, physiotherapy focuses on comfort during ventilation, including bronchial hygiene, passive mobilization, and adjustment of ventilatory support to ensure the least possible discomfort*.” (ICA3) and another added: “...*At home, care is more focused on motor stimulation and sensory input, as well as respiratory care such as suctioning and the use of a manual resuscitator (Ambu bag)*.” (ICB5). 

Figure 1 presents the similarity analysis of physiotherapists’ representations of palliative care approaches in NICU and home settings for neonates. 

**Figure 1:**
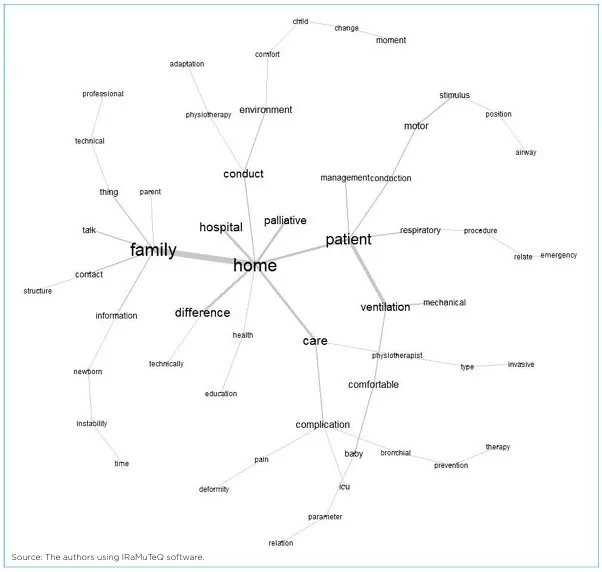
Similarity analysis of physiotherapists’ representations regarding their approaches in NICU and home settings for neonates receiving palliative care.

Source: The authors using IRaMuTeQ software.

 Finally, for category 5, *Challenges and Strategies for Neonatal Palliative Care Implementation*, participants identified challenges in neonatal palliative care, including limited professional recognition in ventilatory management, lack of institutional guidelines, and the emotional burden associated with poor prognoses. In their opinion, “...*Developing a protocol that includes physiotherapists would allow us to work more specifically within neonatal palliative care*.” (ICB4). As strategies for improvement, participants emphasized the need for enhanced professional education, multidisciplinary discussion, and the development of clear institutional protocols defining the role of physiotherapy in neonatal palliative care, as follows: “...*More discussions, more knowledge, and lectures on the topic. Not just general palliative care, but specifically how physiotherapy acts in neonatal palliative care*.” (ICA2) and “...*Information. People have no idea what palliative care is, even inside the hospital*.” (ICB5). Physiotherapists’ experiences highlighted the need for specialized training, better communication and teamwork, clearer definition of professional roles, institutional support for emotional demands, and standardized guidelines to improve the safety, consistency, and quality of neonatal palliative care. 

Figure 2 presents the similarity analysis of physiotherapists’ representations regarding challenges and strategies for optimizing neonatal palliative care. 

**Figure 2:**
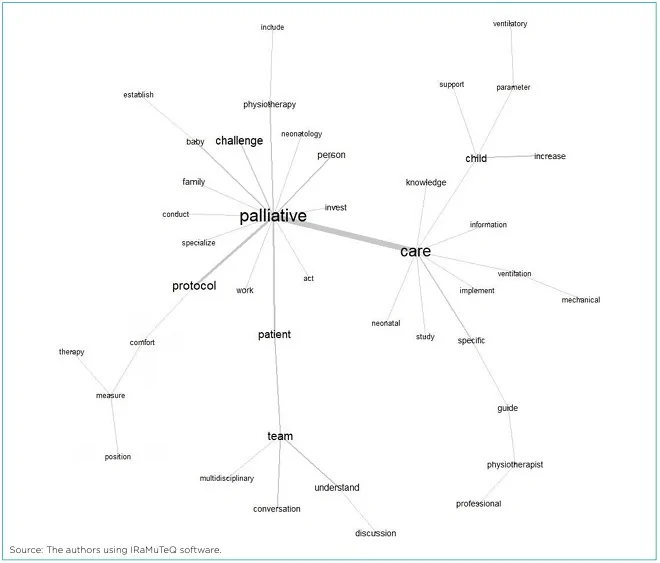
Similarity analysis of physiotherapists’ representations regarding challenges and strategies for optimizing neonatal palliative care.

Source: The authors using IRaMuTeQ software.

## DISCUSSION

 Neonatal palliative care has been increasingly recognized and implemented worldwide, despite ongoing ethical, emotional, and operational challenges, reflecting growing awareness of its importance.^17^ This study is among the first to explore physiotherapists’ knowledge and experiences in neonatal palliative care across two hospitals in different cities, addressing gaps and supporting more effective, humanized, and family-centered physiotherapy practices. 

 Palliative care focuses not only on neonatal comfort but also on support for families. Consistent with previous studies, care should be individualized according to the clinical condition and patient’s responses, prioritizing relief of suffering and emotional support.^18,19^ These challenges are more pronounced in end-of-life situations, as neonatal death disrupts expected life trajectories and complicates acceptance.^20^ The indication for neonatal palliative care represents both a clinical and ethical challenge, requiring careful decisions regarding the limitation of non-beneficial interventions while ensuring comfort, dignity, and effective symptom control for newborns and support for their families.^21^

 Neonatal palliative care involves significant emotional demands related to the suffering of both infants and their families. Physiotherapists reported that their emotional responses were closely linked to parental distress, with some professionals — particularly mothers — describing greater emotional sensitivity when reflecting on maternal suffering. Differences among participants were mainly associated with the intensity of these emotional experiences. These emotional demands are compounded by professional challenges. Physiotherapists frequently reported feeling unprepared to manage patient death, which often leads to frustration and emotional distress. Families, in turn, experience profound suffering as they seek explanations and meaning for the loss while confronting the fragility of life.^22^ In this context, strengthening health professional training, including physiotherapists, is essential to encompass comprehensive end-oflife care, as well as to develop competencies for addressing the complex emotional and existential dimensions inherent to this stage. This also highlights the need for training in relational ethics and narrative communication, which remain insufficiently addressed in most NICUs.^13^

 Effective communication is essential to addressing these challenges and is a core component of therapeutic care, particularly in family involvement, supporting humanized and comprehensive care.^23^ Including families while respecting their values and beliefs enables individualized and ethically grounded care based on beneficence and non-maleficence.^24^ Communication and family interaction may vary according to work conditions. Physiotherapists reported that in the NICU, night shifts can limit direct engagement and continuity of care. In both NICU and home settings, care practices included physical comfort, appropriate positioning, and encouragement of skin-to-skin contact or parental holding, aligning with a family-centered, holistic approach and the importance of parental presence in neonatal care.^25^

 Across both NICU and home settings, physiotherapy contributes to neonatal palliative care by supporting respiratory and motor function and providing individualized guidance to families, aiming to enhance the child’s functional capacity and quality of life. Physiotherapists’ clinical decision-making is an integral part of palliative care; however, evidence on their role remains limited.^26^ These findings highlight the need for structured institutional support, including mandatory training in palliative care, psychological support, and workload adjustments to ensure continuous and compassionate care. The lack of institutional guidelines, particularly regarding patient positioning in critical situations, remains a significant limitation.^27^

 Given these gaps, clearer criteria for identifying newborns eligible for palliative care, along with continuous training and multidisciplinary discussion, are essential to improve care for patients and families. Both institutions already have palliative care initiatives supported by specialized teams, highlighting the need for dedicated neonatal palliative care groups and evidence-based protocols. The growing role of physiotherapists in palliative care, supported by evidence on non-pharmacological interventions, reinforces their contribution within multidisciplinary teams.^28^ Standardized guidelines and staff education promote more consistent care and better end-of-life experiences.^29^ Physiotherapists’ experiences highlight the need for targeted training, improved communication and teamwork, clearer role definition, and institutional support to enhance the quality and consistency of neonatal palliative care. 

 This study has some limitations. Internal validity may have been affected by the inherent subjectivity of participants’ responses, as these reflect individual experiences, perceptions, and interpretations. However, other aspects such as rigorous data collection, systematic coding and categorization of data, triangulation among researchers, and evidence of data saturation enhanced the study’s internal validity. It is also important to acknowledge that the external validity of the findings may be limited by the relatively small, homogeneous sample and the inclusion of only two institutions, which may restrict result transferability to other healthcare settings. However, the participating institutions share organizational and clinical characteristics that are common to many teaching and tertiary care hospitals. Therefore, although caution is needed when generalizing the findings, the results are likely relevant to other institutions with similar structures, patient populations, and physiotherapy practices. 

 Furthermore, since this qualitative study relied on structured interviews, the potential influence of the Hawthorne effect should be recognized. Participants’ awareness of being involved in a research study may have influenced their responses, leading to socially desirable answers or statements aligned with what they perceived to be the researchers’ expectations. However, this potential bias was likely mitigated by the fact that participants were encouraged to share their genuine perspectives, interviews were conducted in a nonjudgmental manner, and questions focused on routine clinical practices and experiences rather than evaluating individual performance. Consequently, although the Hawthorne effect cannot be excluded, its influence on the overall findings is unlikely to have substantially altered the main themes that emerged. 

 In conclusion, physiotherapists demonstrated relevant knowledge of neonatal palliative care; however, the findings identified institutional gaps and training needs. Expanding their participation in palliative care discussions can support the development of institutional guidelines, strengthen professional education, and improve the quality of care. 

## Data Availability

The database that originated the article is available with the corresponding author.
